# Predicting sleep quality with digital biomarkers and artificial neural networks

**DOI:** 10.3389/fpsyt.2025.1591448

**Published:** 2025-07-16

**Authors:** Hyolim Lee, Minsung Cho, Sang Won Lee, Sungkyu Park

**Affiliations:** ^1^ Department of Artificial Intelligence Convergence, Kangwon National University, Gangwon, Republic of Korea; ^2^ Seoul Asan Medical Center, Seoul, Republic of Korea; ^3^ Department of Psychiatry, Kyungpook National University Chilgok Hospital, Daegu, Republic of Korea; ^4^ Department of Psychiatry, School of Medicine, Kyungpook National University, Daegu, Republic of Korea; ^5^ KDI School of Public Policy and Management, Sejong, Republic of Korea

**Keywords:** wearable devices, digital biomarkers, heart rate variability (HRV), sleep quality, artificial neural networks, explainable AI

## Abstract

**Introduction:**

Modern society's increasing stress and irregular lifestyles have led to rising insomnia prevalence, making sleep quality assessment crucial for health management. This study investigates the relationship between heart rate variability (HRV) collected from wearable devices and sleep quality, specifically focusing on wake-after-sleep-onset (WASO) as a critical marker of sleep fragmentation. We aimed to develop predictive models for next-day sleep quality using continuous digital biomarkers.

**Methods:**

We conducted two experiments (winter and summer 2023) with 82 participants who wore Samsung Galaxy Watch Active 2 devices during wakefulness. Biometric data including HRV signals, daily step counts, and physiological indicators were collected alongside subjective questionnaire responses (PHQ-9, GAD-7, ISI, KNHANES, WHOQOL-BREF) and daily sleep logs. We analyzed seven days of preceding data to predict next-day WASO using various machine learning approaches including ARIMA, Random Forest, XGBoost, GRU, TCN, Transformers, and LSTM models.

**Results:**

Among HRV features, the low-frequency to high-frequency (LF/HF) ratio emerged as the strongest correlate with WASO, showing statistically significant differences between groups (Lower LF/HF: 7.5±2.0 min vs. Higher LF/HF: 14.9±3.0 min, p=0.012). LSTM demonstrated superior predictive performance with 90.4% accuracy, 91.3% precision, and 89.9% recall for binary WASO classification. LIME analysis confirmed that LF/HF ratio, along with ISI and WHOQOL-BREF scores, were the most influential features for model predictions.

**Discussion:**

This work introduces a novel approach for managing sleep health through continuous HRV monitoring and predictive modeling using wearable devices. The findings highlight the potential of the LF/HF ratio as a digital biomarker for sleep quality prediction, offering promise for personalized, data-driven healthcare interventions. The superior performance of deep learning methods underscores the value of temporal pattern recognition in sleep quality assessment, paving the way for proactive sleep health management in everyday life.

## 1 Introduction

Modern society, characterized by rapid technological advancements, heightened stress, irregular lifestyles, and excessive workloads, has led to a significant increase in the prevalence of insomnia among individuals (1, 2). According to the American Academy of Sleep Medicine (AASM), approximately 33% to 50% of adults have experienced symptoms of insomnia, with 6% to 10% showing clinically significant symptoms of insomnia (3, 4). Insomnia can considerably affect both an individual’s health and societal well-being. Prolonged sleep deprivation could induce a variety of physical and mental health problems, including chronic fatigue, an increased risk of cardiovascular disease, impaired cognitive performance (e.g., reduced concentration and memory), and depression (5, 6). Furthermore, difficulty sleeping is associated with higher healthcare expenditures than those who never experienced sleep issues, leading to an escalation in societal costs (7). As a result, many efforts have been made to alleviate insomnia symptoms.

Recent studies focus on tracking sleep quality and insomnia using wearable devices, such as smartwatches, to inform potential interventions. Wearable devices can continuously track vital signs, including heart rate (hereafter HR), activity levels, and sleep patterns, as part of a user’s daily routine. This ongoing data collection enables more accurate data-driven sleep analysis (8, 9). Data collected through photoplethysmography (hereafter PPG) sensors, a technology that detects changes in blood flow by shining light on the skin based on the principle that light absorption varies with blood flow during each heartbeat, can provide important physiological indicators, such as heart rate variability (hereafter HRV) (10). HRV reflects stress levels and autonomic nervous system activity and is used as an important indicator to assess the relationship between stress and sleep quality, which allows for the analysis of sleep status (11). Previous research reported that sleep deprivation adversely affects HRV, reflecting autonomic nervous system imbalance. A study showed a decline in HF (high-frequency band) associated with an increase in nLF (normalized low-frequency band) after partial sleep deprivation, indicating a decreased parasympathetic and increased sympathetic activity (12). HRV can be affected by stress and the balance of the autonomic nervous system. A study revealing how stress alters HRV during each sleep stage was conducted on healthy adults. When acute stress was given, levels of the LF/HF ratio increased during NREM sleep, implying a significant decrease in sleep maintenance. In the absence of stress, parasympathetic activity increased, particularly during successive NREM cycles. Whereas acute stress was associated with a decrease in parasympathetic activity. Researchers have suggested the need for further research into the relationships between HRV, psychological stress, and sleep (13).

There are several sleep measurement indices, including wake-after-sleep-onset (hereafter WASO), sleep latency, sleep efficiency, and total sleep time. Among these sleep measurements, WASO is widely recognized as one of the most reliable indicators of sleep quality, particularly in identifying sleep fragmentation and disturbances (14). It measures the total time a person spends awake after initially falling asleep, making it a crucial marker for assessing conditions. As WASO increases, it typically correlates with lower sleep efficiency and overall poorer sleep quality (15). WASO has shown a significant difference between the non-insomnia and insomnia groups. A study by Kristin et al. reported that people with insomnia had higher minutes of WASO than normal people on both sleep diary and actigraphy (16). Other research demonstrated that an increase in WASO was highly correlated with an increase in insomnia symptoms, including sleep maintenance and sleep quality (15).

Furthermore, few studies have researched how night-to-night variability affects sleep measurement indices, including WASO and sleep efficiency. Research conducted by Buysse et al. indicated that chronic insomnia subjects exhibited greater variability in WASO and sleep efficiency compared to non-insomnia subjects. Notably, while no correlation was found with values from the previous night, positive correlations were observed with the values from the two nights prior. However, evidence for positive correlation was weak, suggesting a need to track a greater number of nights to estimate stably. The results suggest that continuous tracking of sleep could enhance insomnia interventions (17).

In this study, we performed a statistical analysis of various HRV characteristics to identify factors that are significantly associated with WASO. Among the features examined, the low-frequency to high-frequency ratio (LF/HF ratio) demonstrated a statistically significant correlation with WASO. Based on this result, we utilized existing predictive models to estimate WASO, leveraging the LF/HF ratio as a key predictor.

The goal of our study is to predict WASO based on HRV data and biosignal data collected through wearable devices. We propose a model to predict the next day’s WASO using HRV data from the previous seven days and investigate the impact of HRV on the assessment of sleep quality. In addition, this study explores the possibility of providing personalized healthcare advice to people with insomnia in the future and aims to present a new paradigm for the management of sleep health through wearable devices.

## 2 Methods

### 2.1 Experimental setup

This study analyzed the relationship between sleep quality and vital sign data by conducting two experiments in 2023. The first experiment was the winter experiment, which lasted 28 days from January 5 to February 1, 2023, and the second was the summer experiment, which lasted 26 days from June 26 to July 21, 2023. All experiments were assessed and approved by the first author’s university’s Institutional Review Board (IRB). A total of 82 participants participated in the experiments, including 24 males and 17 females in the winter experiment and 21 males and 20 females in the summer experiment. The average age of the participants in each experiment was 26.3 ± 6.7 years and 24.2 ± 6.5 years, respectively; in this work, all reported values are in the form of ‘mean ± standard deviation (SD),’ providing a statistical summary of central tendency and variability. Among the 82 participants, 67 were undergraduate students and 15 were non-student adults. All participants were recruited through on-campus advertisements and flyers.

During the experiment, participants wore wearable devices, Samsung Galaxy Watch Active 2, that measured real-time vital signs, such as HR and PPG signals and activity levels. Participants were instructed to remove the smartwatch at bedtime to charge it and to re-wear the fully charged device after waking up; therefore, biometric data were collected exclusively during daytime wakefulness. Participants also periodically completed various clinical mental disorder questionnaires, together with a sleep-related log to rate sleep quality subjectively. The participants’ demographics and the experiment’s timeline are shown in **Table 1**. While the full experiment periods were 28 days (winter) and 26 days (summer), the actual data collection period varied by participant due to individual circumstances and technical issues. In total, 10 participants took part in both the winter and summer experiments.

**Table 1:** Demographics of the participants and the timeline of the experiment.

Experiment	Duration	Participants	Male/Female	Average Age
Winter 2023	28 days (Jan 5-Feb 1, 2023)	41	24/17	26.3 ± 6.8
Summer 2023	26 days (Jun 26-Jul 21, 2023)	41	21/20	24.2 ± 6.5
**Total**	**54 days**	**82**	**45/37**	**25.3 ± 6.7**

Bold is the aggregated value of the two experiments.

### 2.2 System design

**Figure 1:**
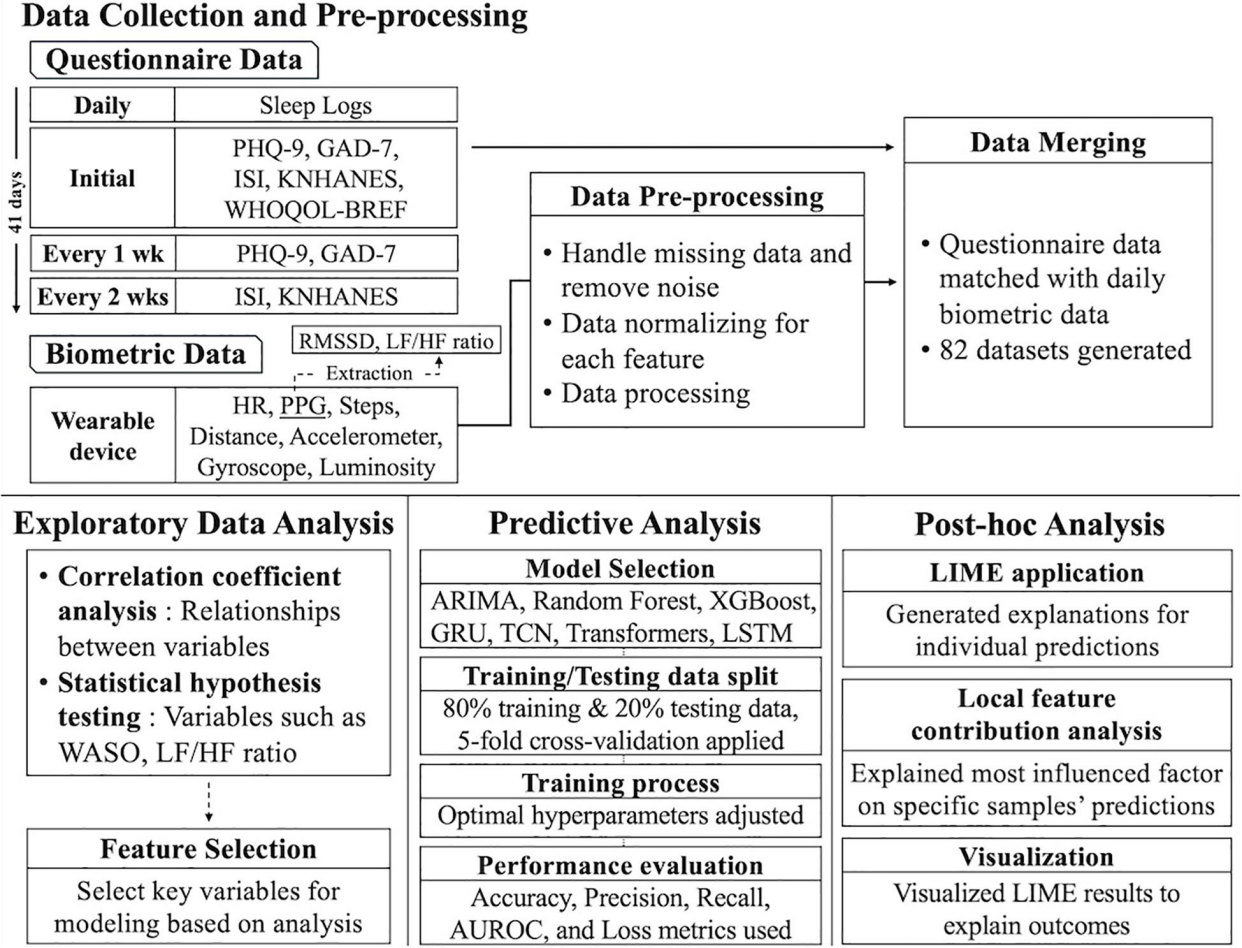
Overall data storage structure. PHQ-9, Patient Health Questionnaire-9; GAD-7, Generalized Anxiety Disorder-7; ISI, Insomnia Severity Index; KNHANES, Stress Questionnaire for Korea National Health and Nutrition Examination Survey; WHOQOL-BREF, World Health Organization Quality-of-Life Brief Version; HR, Heart Rate; PPG, photoplethysmography; RMSSD, Root Mean Square of the Successive Differences; LF/HF, Low Frequency/High Frequency (ratio of HRV metrics); WASO, Wake After Sleep Onset; ARIMA, Autoregressive Integrated Moving Average; GRU, Gated Recurrent Unit; TCN, Temporal Convolutional Network; LSTM, Long Short-Term Memory; and LIME, Local Interpretable Model-Agnostic Explanations.

The system design of this study consists of three phases: data collection, data storage structure, and database structure. **Figure 1** provides a visual representation of the entire process. This structure was designed to manage and analyze various vital signs and questionnaire data efficiently, establishing a comprehensive framework for data integration. It laid the foundation for the researcher to combine and explore the objective vital signs data alongside the participants’ subjective questionnaire data, enabling a holistic approach to data analysis.

### 2.3 Data collection

Data collection can be divided into two parts: collecting biometric data via wearable devices and collecting questionnaire data provided by participants. This dual approach combines objective, quantitative physiological data with subjective, self-reported data to better understand participants’ physical and psychological states.

#### 2.3.1 Biometric data collection

For the biometric data collection process, wearable devices that monitor various physiological signals were used. The data collected included Heart Rate (HR), Photoplethysmography (PPG), Steps, Distance Traveled, Accelerometer, Gyroscope, and Luminosity, each providing insights into participants’ physiological and daily activity patterns. The sampling rates for each biometric signal were as follows: heart rate at 1 Hz, photoplethysmography (PPG) at 10 Hz, acceleration and gyroscope at 50 Hz, and luminosity at 1 Hz.

Heart Rate (HR) refers to the number of beats per minute (BPM) and is an important indicator of cardiovascular activity. It is a direct measure of physical stress and provides real-time insights into a participant’s physiological state (18). For example, heart rate increases during exercise or stressful situations, making it a useful metric for monitoring physical stress levels. Heart Rate Variability (HRV) features were computed based on data collected from the PPG sensor, one of the sensors used in wearable devices. The resulting HRV data serve as a valuable metric for assessing the balance of the autonomic nervous system, which can be used to evaluate stress levels and recovery ability. By analyzing changes between heartbeats, HRV reflects the activity of the sympathetic and parasympathetic nerves (11, 12). Note that all HR and HRV data were computed from the target devices’ same PPG signals using the HeartPy library^1^ in Python.

Steps were used to represent the participant’s daily physical activity level. Distance Traveled was calculated based on the step count and provides valuable information for assessing mobility and overall activity levels. Together, these metrics offer a comprehensive understanding of participants’ physical activity and energy expenditure. Acceleration was derived from the accelerometer sensor and reflects the intensity and frequency of body movements. It can be used to analyze the intensity of different physical activities, such as walking, running, or more vigorous actions. Similarly, the Gyroscope Rate, measured by the gyroscope sensor, captures the angular velocity of movements and is used to analyze posture changes and movement patterns on a per-second basis. Luminosity, measured as illuminance, represents the amount of light in a participant’s environment.

In addition to physiological and activity data, these wearable devices automatically transmit data to a central server every 30 minutes for storage and analysis. This facilitates the examination of relationships between physical activity patterns, environmental factors, and vital signs, allowing for a deeper understanding of participants’ daily lives and overall well-being.

#### 2.3.2 Questionnaire data collection

**Figure 2:**
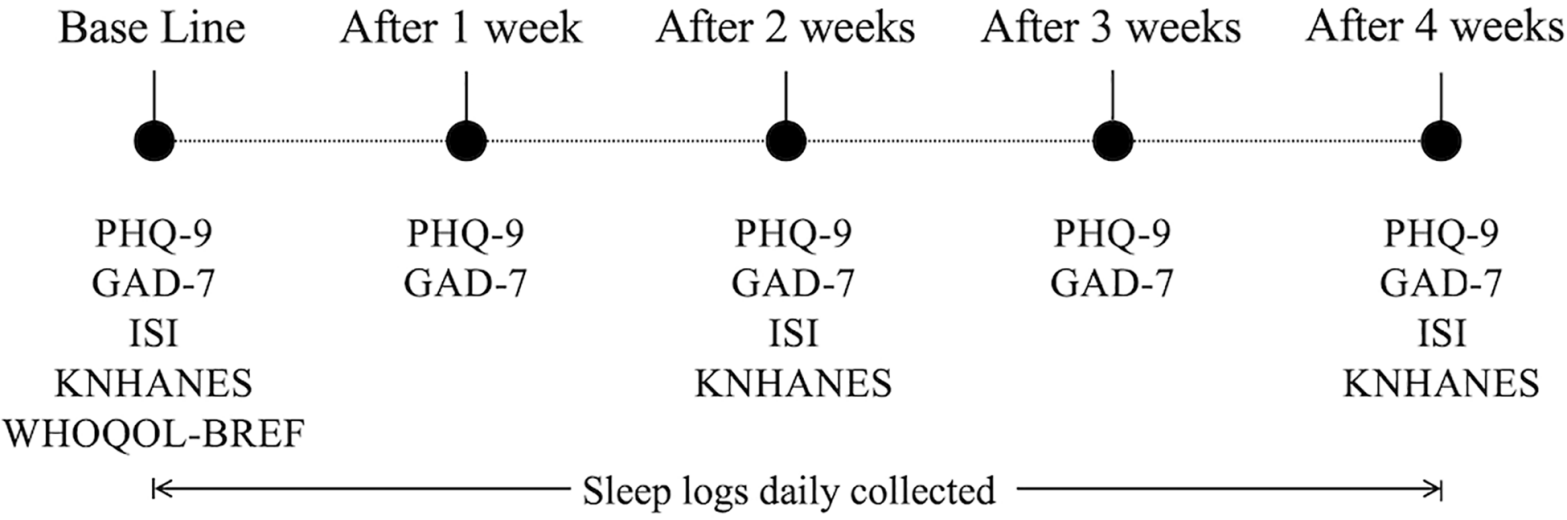
Collection frequency for the self-reported questionnaire data. PHQ-9, Patient Health Questionnaire-9; GAD-7, Generalized Anxiety Disorder-7; ISI, Insomnia Severity Index; KNHANES, Stress Questionnaire for Korea National Health and Nutrition Examination Survey; and WHOQOL-BREF, World Health Organization Quality-of-Life Brief Version.

In addition to the biometric data, participants completed questionnaires at regular intervals throughout the experiment to report their subjective mental states. This questionnaire cycle enabled regular monitoring of the participants’ psychological and physical conditions, as well as their lifestyle patterns, while systematically collecting the data. **Figure 2** shows the weekly protocol of the questionnaires administered, including the frequency of the self-report questionnaires used throughout the experiment.

Specifically, the PHQ-9, GAD-7, ISI, KNHANES, and WHOQOL-BREF were administered during the initial visit. After that, PHQ-9 and GAD-7 were administered weekly, while ISI and KNHANES were administered every two weeks. The data collected from the questionnaires included information on participants’ sleep experiences, including the number and total duration of WASOs as a proxy of sleep quality; the sleep logs were recorded daily. These questionnaire data play an important role in gaining a more comprehensive understanding of the interaction between participants’ mental and emotional states and their vital signs (19). Then, all types of data were unified and stored in a structured database, MongoDB (20) (for the detailed data storage structure, see the Integrated Data and Database Structure section in the **Supplementary Material**).

#### 2.3.3 Data pre-processing

HRV data was extracted every five minutes throughout each day. If a given day contained fewer than 12 valid HRV entries (equivalent to one hour of data), that day was excluded from the analysis to ensure data quality. Only for days with sufficient data, to handle missing values in the time-series data, we applied a structured imputation strategy based on the k-nearest neighbors (KNN) algorithm, which estimates each missing value by averaging the values from the most similar neighbors, i.e., rows composed of five-minute chunks in the case of the HRV features and rows composed of daily chunks in the case of all other features, including activity, sleep, and questionnaires, where those neighbors are decided based on the smallest Euclidean distance among other features except the target one. We empirically set the number of neighbors *k* = 3, considering both performance and data stability. This approach allowed us to preserve signal consistency and avoid potential biases from arbitrary assumptions.

Especially with the HRV features, their imputation for the five-minute chunks impacts relatively less, as they are averaged to be daily chunks in the forthcoming analysis. For sleep logs, we confirm that there are fewer missing values (see **Supplementary Figure S4**) as we daily monitored during the experiment and asked 82 participants to answer if they had not input sleep logs within a certain period. To prevent data leakage, all imputation steps were strictly performed on the training and testing sets separately.

### 2.4 Statistical analysis

To analyze the relationship between WASO and HRV, we divided the LF/HF ratio data, one type of HRV variable, based on a cutoff value of 0.58; this value was set based on the median of the LF/HF ratio across all participants to achieve a balanced grouping. As a consequence, the participants were divided into two groups: Lower LF/HF ratio (n=40, 0.5 ± 0.1) and Higher (n=42, 0.7 ± 0.1). The LF/HF ratio, an established indicator of autonomic nervous system balance, reflects the interplay between sympathetic (low frequency) and parasympathetic (high frequency) activities (21). A lower ratio indicates parasympathetic dominance, while a higher ratio reflects sympathetic dominance.

We then analyzed whether there was any difference between the LF/HF ratio-based groupings on WASO. After checking the distribution of each feature using the Kolmogorov-Smirnov (K-S) test, we found that both WASO and LF/HF ratios deviated from a normal distribution (*p <* 0.05). Based on these results, we used a non-parametric approach, the Wilcoxon Rank-Sum Test.

Next, we performed a Pearson correlation coefficient analysis to analyze the correlation between the biosignals collected in the experiment. The correlation matrix (see the **Supplementary Figures** for the entire correlation matrix) provides a visual representation of the correlation between each biosignal, allowing us to address multicollinearity. If the correlation coefficient was relatively high, it was considered to contribute to redundancy in representation or potentially degrade model performance and was excluded as a feature. After excluding the strongly correlated signals, the remained ones were selected to be used as input variables for the forthcoming machine-learning model.

### 2.5 Modeling methods for predictive analytics

Based on the correlation coefficients and variance inflation factor (VIF) analysis (22), we selected a range of features (*X*) to predict WASO (*y*) of the next day. These features were carefully selected based on their correlation with the target variable (*y*) and with the LF/HF ratio, as well as their VIF scores, to minimize multicollinearity. Variables with VIF values greater than five were considered to indicate multicollinearity, and we implemented procedures to ensure that these variables were not included as features (i.e., independent variables) in the modeling process. To prevent data leakage, we performed feature selection process within the training/testing cross-validation folds using a nested cross-validation approach. This ensures that test data does not influence feature selection, enhancing model validity and reliability (23).

The modeling process leveraged the most appropriate combinations of features to ensure optimal predictive performance. For the prediction of WASO, we employed a variety of time-series analysis models and machine learning models, including Autoregressive Integrated Moving Average (ARIMA), Random Forest, XGBoost, Gated Recurrent Unit (GRU), Temporal Convolutional Network (TCN), Transformers, and Long Short-Term Memory (LSTM).

Each model was chosen based on the characteristics of the data and its unique strengths for predicting WASO. ARIMA is widely used for analyzing and forecasting time-series data, excelling at capturing trends and seasonality effectively, which is crucial for modeling temporal changes in WASO (24). Random Forest is an ensemble model capable of learning complex non-linear relationships while being robust to overfitting, making it suitable for handling diverse feature sets (25). XGBoost is a high-performance machine learning model that improves on the Gradient Boosting algorithm, providing fast learning speed and high prediction performance. It combines multiple weak learners based on a Decision Tree to create a strong predictive model (26). GRU, a type of recurrent neural network, efficiently captures long-term dependencies in sequential data with lower computational cost compared to other RNN variants (27). TCN leverages convolutional layers to model temporal dependencies in a highly parallelized manner, enabling efficient handling of time-series data (28). With their attention mechanisms, the Transformers model captures complex relationships within long sequences, offering flexibility in modeling intricate temporal patterns (29). Lastly, LSTM networks are designed to learn long-term dependencies and are particularly effective at modeling the persistent patterns in time-series data, making them a natural fit for WASO prediction (30).

The target variable, WASO, was categorized into two classes: 0 (Lower WASO) and 1 (Higher WASO). This binary classification was established due to the nature of sleep interruption. A WASO value of 0 indicates that the subject had uninterrupted sleep with no awakening episodes during the night, representing ideal sleep quality. On the other hand, any non-zero WASO value (i.e., 1) indicates certain degrees of wakefulness during the night (Min value: 2 min, Max: 38 min, Mean: 13.1 min; see **Supplementary Figure S2** for the frequency distribution). The distribution of the two classes is as follows: 0 (Lower WASO) accounts for 41.5%, while 1 (Higher WASO) represents 58.5%. Since the data is relatively evenly distributed between the two classes, it is appropriate to use the dataset as-is for classification modelling without additional adjustments for class imbalance.

For the problem formulation, data from *t* −6 days to *t* day (a total of seven days) were used to predict the WASO on *t* +1 day. Fixing seven days as a window size and moving windows with *stride*=1 means that each user from the first (winter) experiment is represented by 22 sets of feature vectors (28 days – 7 win size + 1 = 22) and 20 sets (26 - 7 + 1 = 20) from the second (summer) experiment. The total number of possible input feature vectors (*X*) for the model is 1,722 (41 users × 22 sets + 41 users × 20 sets = 1722, note that we got WASO (*y*) until the day after the experiment, so we could use until the very last input feature vector for each subject) with *batch size*=32. This temporal window was selected to capture a full weekly cycle, as modern people often exhibit similar behavioral patterns on a weekly basis (31). Specifically, the inclusion of seven days ensures that the model incorporates weekend data, which may differ significantly from weekday patterns. People’s activity levels and sleep routines can vary between weekdays and weekends, so using a full week’s worth of data allows for more accurate modeling of these cyclical patterns in sleep behavior and their influence on wakefulness.

In summary, we tried to predict WASO using ARIMA, Random Forest, XGBoost, GRU, TCN, Transformers, and LSTM, with input data spanning from *t* −6 days to a *t* day. The binary classification of WASO, 0 (Lower WASO), and 1 (Higher WASO) was chosen based on its practical relevance, as it differentiates between uninterrupted and disturbed sleep. For ARIMA, classification was performed by setting a threshold based on the ROC curve, allowing us to determine the optimal cutoff point for distinguishing between the two classes. The use of a full week’s worth of data enhances the model’s ability to capture weekend effects, which may differ from weekday patterns.

## 3 Results

### 3.1 Comprehensive data overview

**Figure 3:**
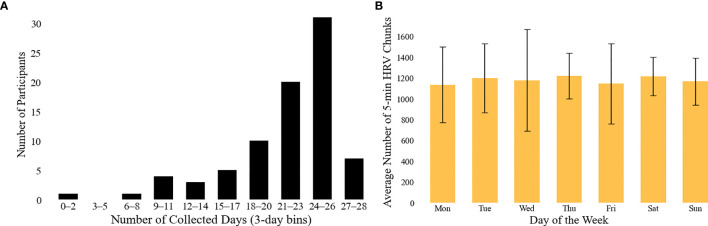
Overview of HRV data availability. **(A)** illustrates the distribution of the number of valid days per participant contributing HRV data, binned in 3-day intervals. Note: the maximum number of valid days was 28 for the first experiment and 26 for the second; only days with at least one hour of HRV data (i.e., 12 or more five-minute samples) were considered valid. **(B)** presents the average number of valid 5-minute HRV chunks collected per day across the week. Error bars indicate the standard deviation across subjects.

In the current work, experiments were conducted to monitor participants’ physiological, physical activity, and psychological states. The number of days of data collected per participant is summarized in **Figure 3**; (A) the majority of the participants participated on most days of the experiment, and (B) the average number of the retrieved five-minute HRV chunks is relatively steady across a week, so we conjecture less bias based on a certain day of the week. Data collected from wearable devices and self-reported questionnaires were analyzed to provide insights into participants’ daily patterns and overall health status. The results are summarized in **Table 2**.

**Table 2:** User biometric and questionnaire data with Mean ± SD values and data counts.

Data Type	Description	Mean ± SD (n = 82)	Count
Through smartwatch (passive data)			
HR	Daily average of heart rate, calculated based on data collected every second during wakefulness, excluding sleep intervals.	83.4 ± 11.2 (*bpm*)	2,214
RMSSD	Daily average of RMSSD, calculated from 5minute intervals based on PPG data collected at 10 Hz, limited to periods of wakefulness.	135.4 ± 37.9 (*ms*)	2,214
LF/HF Ratio	Daily average of LF/HF ratio, derived from frequency-domain analysis of HRV data using PPG, limited to periods of wakefulness.	0.6 ± 0.2 (*ms*)	2,214
Steps	Daily cumulative step count, calculated from step data recorded every second, excluding sleep periods.	4953.8 ± 10200.8 (*step*)	2,214
Distance	Daily cumulative distance traveled, derived from data collected every second using a pedometer, excluding sleep periods.	3746.8 ± 7673.8 (*m*)	2,214
Accelerometer	Daily average of acceleration, computed from data collected every second, restricted to wakeful periods.	X: 1.2 ± 34.9 Y: -4.0 ± 24.9 Z: 3.6 ± 34.5 (*m/s* ^2^)	1,621
Gyroscope	Daily average of rotational movement, computed from gyroscope readings collected every second, excluding sleep intervals.	X: -0.2 ± 36.7 Y: -0.0 ± 18.7 Z: -0.2 ± 26.3 (*deg/s*)	1,616
Luminosity	Daily average of brightness level, calculated from ambient light sensor readings collected every second, excluding sleep intervals.	716.5 ± 1023.0 (*lux*)	2,214
Through self-reported survey (active data)			
Questionnaire	Weekly responses from five questionnaires (PHQ-9, GAD-7, ISI, KNHANES, WHOQOL-BREF), providing insights into participants’ psychological and behavioral states.	PHQ-9: 1.7 ± 2.6 GAD-7: 1.1 ± 2.3 ISI: 5.8 ± 4.2 KNHANES: 28.7 ± 10.3 WHOQOL-BREF: 99.4 ± 13.3 (*score*)	1,394
Sleep Log	Daily records of wakefulness after sleep onset (WASO)	13.1 ± 6.5 (*min*)	2,296

Data collection times are specified, with measurements taken only during on-wrist periods. Participants removed the smartwatch at bedtime to charge it and re-wore the fully charged device after waking up; All units are daily-basis except for the questionnaire responses; Biometric data were collected through a smartwatch, while self-reported survey data were submitted by participants via a web-based interface.

The physiological data included Heart Rate (HR), Root Mean Square of the Successive Differences (RMSSD), and the LF/HF ratio. The daily average HR was 83.4 ± 11.2 *bpm*, reflecting participants’ baseline cardiovascular activity. RMSSD, an indicator of parasympathetic nervous system regulation, had a daily average of 135.4 ± 37.9 *ms*. The LF/HF ratio, which assesses the balance between sympathetic and parasympathetic nervous system activity, was measured at 0.6 ± 0.2 *ms*. Physical activities included steps and distance traveled data. The daily cumulative step count was 4953.8 ± 10200.8 *steps*, while the daily cumulative distance traveled was 3746.8 ± 7673.8 *meters*, both reflecting participants’ mobility and physical activity levels. The statistics of the full features, such as acceleration, gyroscope for physical activities, and luminosity for environmental traits, are listed in **Table 2**. Self-reported questionnaire data provided valuable insights into participants’ psychological and behavioral states. The mean scores for each questionnaire were as follows: PHQ-9 at 1.7 ± 2.6, GAD-7 at 1.1 ± 2.3, ISI at 5.8 ± 4.2, KNHANES at 28.7 ± 10.3, and WHOQOL-BREF at 99.4 ± 13.3. These scores reflect variations in mental health and overall quality of life among participants. Lastly, the sleep log data captured participants’ wakefulness after sleep onset (WASO), with an average of 13.1 ± 6.5 minutes per day. This metric provides a quantitative assessment of sleep quality and fragmentation. Also, we visualize the weekly distribution of the LF/HF ratio and WASO features to depict potential differences in weekdays and weekends (see **Supplementary Figure S5**); while LF/HF ratio is relatively steady, the WASO values tend to be greater on weekends.

### 3.2 Exploratory data analysis

**Figure 4:**
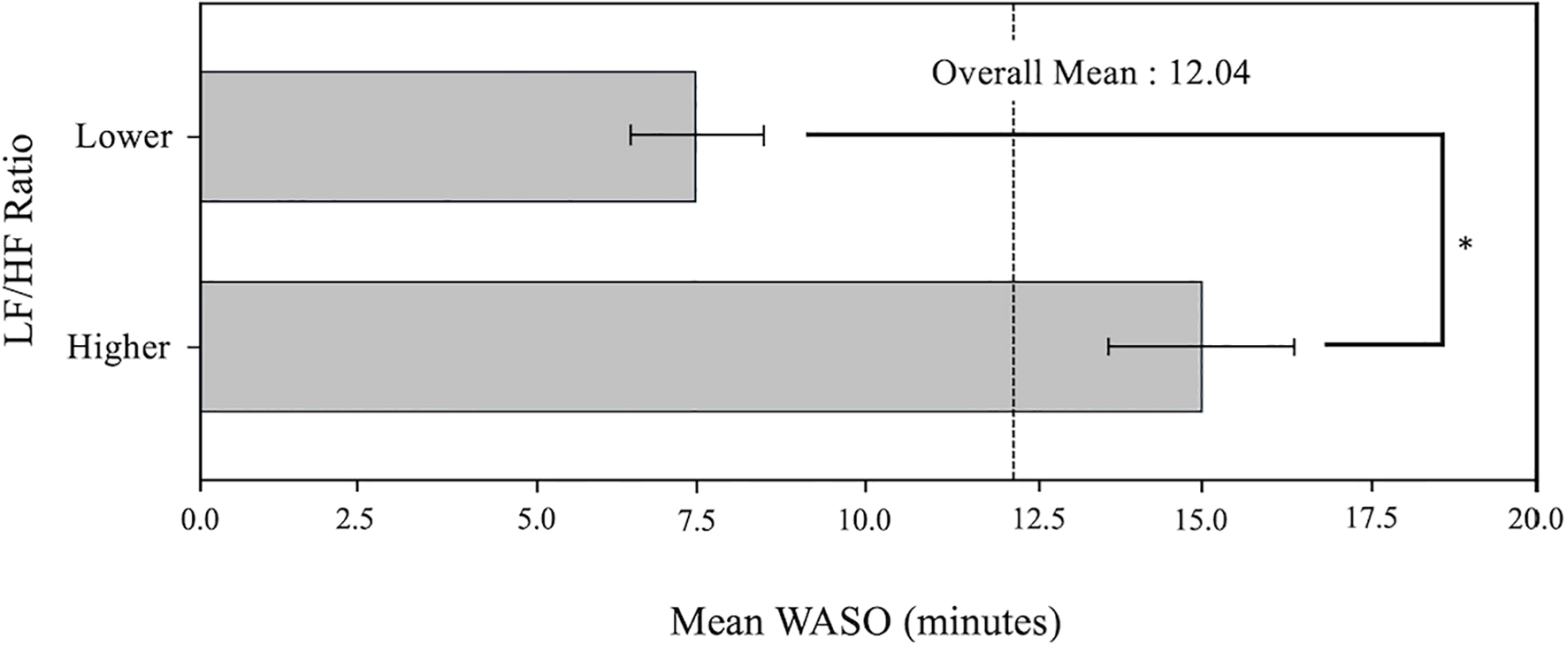
Mean WASO by LF/HF ratio groups. The error bar stands for standard deviation.

The Wilcoxon Rank-Sum Test revealed a statistically significant difference in the distribution of WASO between groups categorized by the LF/HF ratio (Lower LF/HF ratio: 7.5 ± 2.0 min and Higher LF/HF ratio: 14.9 ± 3.0 min with *W* = −5.0*, p* = 0.012).**Figure 4** depicts the mean WASO values for each LF/HF ratio group (Lower, Higher). The bars represent the mean WASO value for each group, and the error bars show the standard deviation, providing a visual indication of the data variability between groups.

The Lower group had a lower WASO than the Higher group, suggesting that the group with a lower LF/HF ratio has better sleep quality. The dashed line represents the average WASO value across all participants, highlighting the difference between the two groups.

We then check the pairwise correlation coefficients across features to reduce the complexity of the prediction model. For instance, the correlation analysis revealed a relatively high correlation between distance traveled and steps, with a correlation coefficient of about 0.3. This suggests that the two variables are likely to provide some similar information. As a consequence, steps were selected and included in the list of independent variables. To further filter out features, we set up a threshold of the correlation coefficient of 0.2 and discarded one of the pairs that exceeded the threshold. As a consequence, a few features, including distance traveled and HR, were dropped.

### 3.3 Predictive analytics

Based on the significant results from the LF/HF ratio analysis in the previous section, we extended our analysis to include additional features for predictive modeling. Since LF/HF ratio was correlated with WASO (*r* = 0.22*, p <* 0.05; **Supplementary Figure S6** also presents a scatter plot of continuous WASO values against LF/HF ratio to illustrate their relationship, including a regression line with *R* ^2^ = 0.23), we wanted to use LF/HF ratio as a core feature and evaluate its interaction with other physiological variables to improve the model’s predictive capabilities. The goal was to explore which other variables, when combined with LF/HF ratio, could improve the accuracy of WASO prediction.

#### 3.3.1 Optimal variable set via correlation coefficients and VIF analysis

After the filtering process from the correlation analysis, we further checked for multicollinearity issues by performing a VIF analysis. Based on the VIF analysis results, the optimized variable combinations were set according to three criteria: correlation coefficients less than 0.1, 0.2, and 0.3, each consisting of a specific combination of variables. In the *<* 0.1 correlation criterion, two combinations were set: the first combination *A*_1_ included LF/HF ratio, and steps variables; and the second combination *A*_2_ included RMSSD, steps, light level, and GAD-7 variables. These combinations were constructed so that the contribution of each variable could be analyzed independently while the correlation between the variables was very low. Predictions were made from different aspects, with combination *A*_1_ using the LF/HF ratio, which represents the balance of the autonomic nervous system, and combination *A*_2_ using RMSSD, which reflects the stability of the autonomic nervous system.

Combinations *B*_1_, *B*_2_, and *B*_3_ were set based on a correlation coefficient of less than 0.2. Combination *B*_1_ included LF/HF ratio, RMSSD, steps, acceleration, gyroscope, and WHOQOL-BREF; combination *B*_2_ included LF/HF ratio, RMSSD, steps, acceleration, gyroscope, ISI, and WHOQOLBREF variables; and combination *B*_3_ included LF/HF ratio, RMSSD, steps, acceleration, gyroscope, PHQ-9, and KNHANES variables. These combinations aimed to provide a more comprehensive prediction of awakenings during sleep by combining physiological and physical activity data and to further analyze the psychological factors of awakenings by including psychological variables such as anxiety and insomnia.

Finally, based on a correlation coefficient of less than 0.3, we constructed a single combination *C* that included LF/HF ratio, RMSSD, steps, acceleration, gyroscope, GAD-7, ISI, and WHOQOL-BREF variables. This combination was an approach to increase the precision of the prediction model by covering as many different vital signs and psychological factors as possible. Although the correlation coefficient may be somewhat high, the intention was to maximize the performance of the WASO prediction by incorporating more variables. Based on these six variable combinations, we evaluated the contribution of each variable to WASO prediction. From this analysis, we selected the optimal combination of variables for modeling.

#### 3.3.2 Predictive modeling results

Based on the combination of variables (*A*_1_ *,A*_2_ *,B*_1_ *,B*_2_ *,B*_3_, and *C*), we applied various machine learning models to evaluate the performance of the WASO prediction model. The models used were Autoregressive Integrated Moving Average (ARIMA), Random Forest, XGBoost, Gated Recurrent Unit (GRU), Temporal Convolutional Network (TCN), Transformers, and Long Short-Term Memory (LSTM). Since binary classification is impossible for ARIMA models, we used ROC curves to determine the optimal threshold and then performed classification. The performance of each model was evaluated based on Accuracy, Precision, Recall, AUROC, and Loss. This performance evaluation aimed to understand how much each combination of variables contributes to the prediction and optimize the WASO prediction (see the **Supplementary Tables** for prediction of other target variables *y*).

Among the various combinations of variables, we present the results for Combination *B*_2_, as it demonstrated the best overall performance across all evaluation metrics. The results for the second best performing combination *A*_1_ can be found in the **Supplementary Tables**. We compared the performance among other prediction models based on *B*_2_ as presented in **Table 3**. The Random Forest model had an accuracy of 0.846 and provided stability in prediction by evaluating a wide range of variables. The XGBoost model performed favorably in predicting specific wakefulness states, with an Accuracy of 0.848 and Precision of 0.860, indicating that the XGBoost model is strong at reducing unnecessary false positives. The Gated Recurrent Unit (GRU) model had an accuracy of 0.831, lower than the LSTM. Still, its simple structure and low computational cost make it suitable for real-time prediction. The Long Short-Term Memory (LSTM) model performed best in Combination *B*_2_, with an Accuracy of 0.904, Precision of 0.913, and Recall of 0.899. The combination *B*_2_ includes LF/HF ratio, RMSSD, steps, acceleration, gyroscope, ISI, and WHOQOL-BREF, and comprehensively considers autonomic nervous system and physical activity data and psychological factors. By combining these variables, the LSTM model was able to accurately predict wakefulness during sleep by effectively reflecting time-dependent physiological changes.

**Table 3:** Performance of the various models to predict WASO (as a binary classification) based on the independent variable combination *B*_2_.

Model	Accuracy	Precision	Recall	AUROC	Loss
ARIMA	0.810	0.822	0.809	0.781	0.401
Random Forest	0.846	0.851	0.840	0.851	0.419
XGBoost	0.848	0.860	0.842	0.866	0.423
GRU	0.831	0.813	0.809	0.802	0.455
TCN	0.832	0.826	0.810	0.811	0.322
Transformers	0.843	0.820	0.801	0.834	0.399
LSTM	**0.904**	**0.913**	**0.899**	**0.901**	**0.263**

Bold is the best value, and underlining is the second best. The classification threshold was determined based on ROC curve analysis.

Moreover, the physical activity level can be a potential confounder, as our experiment allowed participants to do anything freely. To address this, we stratified participants into three groups based on their average daily step counts: Low (n=27, 3314.4 *steps* in average), Middle (n=28, 5005.1 *steps*), and High (n=27, 7452.7 *steps*). We then compared model performance across these groups using the same evaluation process with our main model; for each group, we divided the data into 8:2 for train and test, then we trained and tested the LSTM model. The first to the third rows of **Table 4** summarize that the prediction performance showed marginal variation across groups. These results may indicate that the physical activity level, as quantified by steps counted, did not significantly affect the model performance, and our model is robust across different activity levels.

**Table 4:** First-third rows: Performance of the different steps groups to predict WASO (as a binary classification) based on the independent variable combination *B*_2_ and the LSTM model.

Group	Accuracy	Precision	Recall	AUROC	Loss
Low	0.899	0.931	0.908	**0.905**	**0.249**
Middle	0.890	0.895	**0.914**	**0.905**	0.271
High	0.885	**0.932**	0.912	0.889	0.250
Weekday	0.820	0.845	0.780	0.825	0.300
Weekend	0.800	0.825	0.790	0.805	0.310
Our model	**0.904**	0.913	0.899	0.901	0.263

Fourth-fifth rows: Performance of the different weekday-weekend groups based on the same evaluation setting. Bold is the best value, and underlining is the second best. The classification threshold was determined based on ROC curve analysis.

Additionally, to account for temporal behavioral differences, we conducted a subgroup analysis by training separate models for weekday and weekend data and evaluating separate models using the same feature set and model architecture but different input window size (*X*), such as 5 (Monday to Friday) for the weekday model trained to predict WASO on Saturday night and 2 (Saturday to Sunday) for the weekend to predict Monday night; since the number of weekdays was larger than that of weekends (each weekday occurred eight times during the study period, while each weekend day occurred seven times), we randomly sampled workdays (evenly from Monday to Friday) to be the same volume of weekends. As presented in the fourth to the fifth rows of **Table 4**, we found that as variable distributions differ, the overall model performance of weekdays was slightly better than that of weekends, although their performance was worse than other results in the same table, maybe linked to the relatively small input window size.

#### 3.3.3 Explainable LIME analysis and forecast results

**Figure 5:**
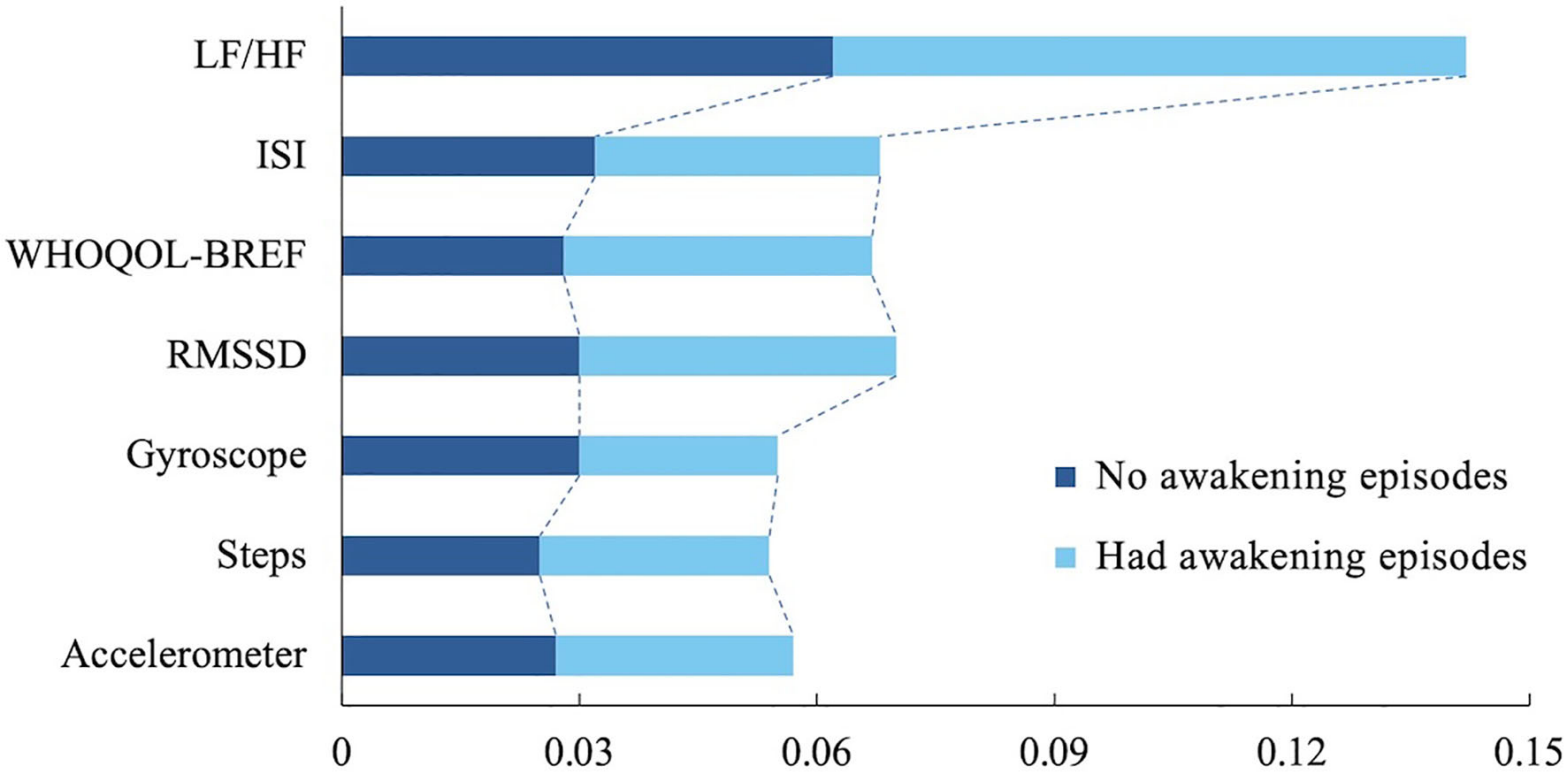
LIME analysis depicts important features contributing to the model’s inference. LF/HF, Low Frequency to High Frequency ratio (one of HRV metrics); ISI, Insomnia Severity Index; WHOQOLBREF, World Health Organization Quality-of-Life Brief Version; and RMSSD, Root Mean Square of the Successive Differences.

To increase the interpretability of the model, we analyzed the predicted WASO results based on the combination *B*_2_ using the Local Interpretable Model-Agnostic Explanations (LIME) technique. The LIME analysis provides a visual representation of which variables were dominant when the model made a particular prediction, allowing researchers to better understand the model’s decision-making process. **Figure 5** shows the results of the LIME analysis, highlighting that the LF/HF ratio was a key predictor, suggesting that the balance and stability of the autonomic nervous system influence wakefulness during sleep. Additionally, the ISI metric for insomnia and the WHOQOL-BREF for quality of life were also found to be significant variables. ISI metrics reflect the impact of a user’s insomnia symptoms on wakefulness during sleep, and the LIME analysis showed that WASO values tended to increase with higher levels of insomnia, indicating that more severe insomnia symptoms are likely to result in longer wakefulness during sleep. The WHOQOL-BREF metrics also showed that a user’s overall life satisfaction is an important psychological factor that affects sleep quality. Suppose the WHOQOL-BREF metric is low. In that case, the user’s stress level may be higher, or quality of life may be lower, resulting in more frequent awakenings during sleep, which was visually confirmed by the LIME analysis in **Figure 5**. These findings suggest that physiological indicators (LF/HF ratio) and psychological indicators (ISI, WHOQOL-BREF) interact to play an important role in predicting awakening times during sleep. In particular, ISI and WHOQOL-BREF indicators, in addition to the autonomic nervous system and physical activity data, contributed to explaining the impact of psychological state on sleep quality. As a result, the model was able to predict users’ sleep status more accurately, providing useful information for developing personalized sleep management and intervention strategies.

## 4 Discussion

About the daily average heart rate, 83.4 *bpm*, the value appears to be slightly higher compared to a previous study using ECG (32). However, our data were collected not only during resting conditions but also during daily activities, such as walking or exercising. A recent study using PPG signals reported real-world heart rate norms in healthy individuals (33); in that study, the median value of the average heart rate in individuals in their early 20s was around 80 bpm, which is similar to our findings.

We then showed that the low and high groups of the LF/HF ratio are significantly different in terms of the extent of the WASO. The result suggests that the dominance of the sympathetic nervous system is strongly associated with increased wakefulness during sleep, resulting in poorer sleep quality. In contrast, in the Lower group, where the parasympathetic activity is relatively dominant, wakefulness is shorter, indicating better sleep quality. Based on this statistical testing, we conducted predictive analyses, and as a consequence, the LF/HF ratio emerged as a significant HRV variable predicting WASO among healthy subjects. Individuals with a low LF/HF ratio showed lower WASO compared to those with a high LF/HF ratio. Our results suggest that an increase in the LF/HF ratio, reflecting greater dominance of the sympathetic activity, may result in poorer sleep quality. Additionally, incorporating the LF/HF ratio alongside self-reported symptom measures, such as the ISI, could enhance the predictive power of the model. These findings indicate that certain HRV measures, such as the LF/HF ratio obtained through continuous monitoring of PPG signals, could be used to predict sleep quality metrics like WASO.

For the predictive modeling results, recurrent neural networks-based LSTM showed the best performance across all metrics (**Table 3**) compared to more conventional time series analysis methods, such as ARIMA, as well as conventional machine-learning methods, such as Random Forest and XGBoost. In the current study, the deep-learning approach showed supremacy aligned with the results from other similar studies (34). However, due to the black-box nature of the deep-learning methods, we further used an explainable method, LIME, and presented that the LF/HF ratio was more crucial than other features – including questionnaires, other HRV features, and sensor data from the wearable devices – in inferring future sleep quality.

The results of the LF/HF ratio could be interpreted in two ways: direct and indirect. Although some controversies exist, the LF/HF ratio is commonly used as a measure of sympathetic to parasympathetic autonomic balance (21, 35, 36). Therefore, an increased LF/HF ratio could, firstly, be related to stressful events in daily life, which might indirectly affect sleep quality. Many studies have shown that chronic stress increases sympathetic activity and decreases parasympathetic activity, implying autonomic imbalance (3, 13, 37). Acute stress reduces parasympathetic activity during NREM and REM sleep, impairing sleep maintenance while increasing sympathetic activity and the LF/HF ratio during NREM. In contrast, the non-stress group shows enhanced parasympathetic activity (13). A recent study continuously using portable electrocardiography reported a relationship between subjective well-being and the LF/HF ratio (38). Additionally, sleep-related stressors, such as sleep deprivation, could be associated with the LF/HF ratio. A previous study showed that sleep deprivation leads to a decline in HF, associated with an increase in LF (12). In another study by Holmes et al., acute sleep deprivation led to an increase in sympathetic activity (39). However, interpretation should be cautious as several studies have reported different results (40, 41). Secondly, there was a direct relationship between HRV metrics, particularly LF/HF ratio, and WASO, although the evidence is limited. A previous study assessed the autonomic nervous system changes in participants with increased sleep onset latency and WASO, respectively. The normalized LF and LF/HF ratio increased in the longer WASO group, while normalized HF decreased. Participants with longer sleep onset latency showed higher normalized LF, particularly in young adults. These findings suggest that the autonomic nervous system can serve as a predictive marker for sleep indices related to sleep onset and maintenance (42).

In this study, the LF/HF ratio showed the most significant correlation with WASO, followed by the ISI and WHOQOL-BREF. The ISI is one of the most reliable and widely used indices for evaluating the severity of insomnia. It enables clinicians to diagnose insomnia using brief questions (43). However, the ISI was not the most significantly correlated variable in our study, and we believe there are several reasons for this. First, daily continuous HRV metrics related to the autonomic nervous system were more appropriate for predicting the next day’s WASO compared to weekly self-reported measurements, such as the ISI. Second, the ISI is more appropriate to predict sleep efficiency rather than WASO. Previous studies reported a moderately positive correlation between WASO and ISI, which was weaker than the correlation with sleep efficiency (44, 45).

Meanwhile, we acknowledge some weaknesses in the current work. First, since each feature has a different granularity, e.g., HRV features are aggregated daily while self-reported questionnaire data are gathered weekly or bi-weekly, the same values from questionnaire data are injected into the daily predictive models and may affect the predictive performance. Second, our subjects reported only a few sleep problems. For example, the low LF/HF ratio group showed shorter WASO (7.5 min) compared to the high LF/HF ratio group with longer WASO (15.0 min), but the overall mean value (13.1 min) was lower than the most commonly used WASO cutoff value (30.0 min) (42, 46). It indicates that the sleep traits of the subjects in our study were relatively healthier than those of the sleep disorder sufferers. Because of this distribution, we set the prediction problem formulation not as regression but as classification because a small value difference of WASO among healthy participants does not matter much in the current study. Third, it is hard to generalize our prediction results. It is because, also related to the second point, the cohort in the current study is young and healthy, lacking diversity and the full range of WASO and HRV. In that sense, we can propose future hypotheses based on our outcomes, such as considering the relationship between the LF/HF ratio and WASO in older individuals. In general, both the LF/HF ratio (47) and WASO (48) increase with age, and therefore, the relationship between the LF/HF ratio and WASO may vary across age groups. Last, in the current work, the sleep-related features, including WASO, are retrieved manually rather than through polysomnography or actigraphy. Therefore, human bias or subjectivity may be a confounding factor in our study (49). In that sense, we would expand the work by recruiting more diverse participants and sensing sleep data more passively from wearable devices.

## 5 Concluding remark

Unlike conventional short-term HRV features retrieved under lab or clinical settings, it has been reported that continuous HRV is capable of capturing aggregated levels of psychological traits such as daily wellbeing or mood (38, 50). To the best of our knowledge, this is the first line of work to utilize continuous HRV to learn and predict sleep quality. We first explored the associations between HRV and sleep features and then successfully inferred the next day’s sleep quality (WASO) with an accuracy of 90.4% in terms of the binary classification setting. We shed light on how continuous monitoring of HRV features can help enhance the quality of sleep in everyday life.

## Data Availability

The raw data supporting the conclusions of this article will be made available by the authors, without undue reservation.

## Footnotes

1 HeartPy. https://python-heart-rate-analysis-toolkit.readthedocs.io/en/latest/.
