# Supplementary material for: Predicting sleep quality with digital biomarkers and artificial neural networks
Source: Front Psychiatry. 2025 Jul 16;16:1591448. doi: 10.3389/fpsyt.2025.1591448 (PMC12308496; doi:10.3389/fpsyt.2025.1591448)
Supplement: Supplementary file 1 [file DataSheet1.pdf]

Supplementary Material

1 INTEGRATED DATA AND DATABASE STRUCTURE

The collected data is structured and stored by a participant, with each user's data organized by date in a CSV file format, facilitating easy access and analysis for researchers. In this study, MongoDB is used to manage data, with each collection containing different data types and organized by user ID for data management. The system design of this study is structured into three phases: data collection, data storage structure, and database structure. Figure S.1 visually represents the overall system structure, illustrating how each component integrates to support efficient data management and analysis.

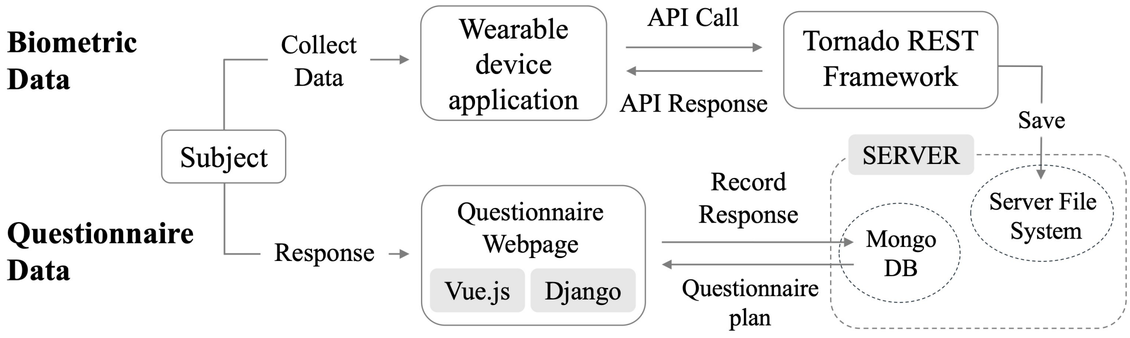

Figure S.1: The overview of the data storage structure.

The database is organized into three collections: one for storing user information, one for questionnaire data, and one for managing questionnaire plans. The first collection, which stores user information, contains each participant's ID and serves as a reference point to link with other data in the database. This enables the organization of each user's data, making identifying and tracking user-specific data easier. The second collection stores the responses to the questionnaires provided by users. This collection organizes subjective questionnaire data by date, enabling the retrieval of responses at specific points in time. For example, questionnaire data collected on a particular day is stored alongside the corresponding user activity information. The third collection manages, stores, and oversees the questionnaire schedule assigned to each user. This collection tracks the questionnaire items that users must submit during specific periods. These are managed by the user, allowing for easy tracking of submitted questionnaires at specific times and helping to prevent missing data.

This database structure streamlines data management and accessibility with a clear division of roles among collections. Additionally, MongoDB's flexible data enables easy adaptation to changes or expansions in the data that may arise during research, allowing for quick access to information as needed. It was designed for the management and comprehensive analysis of objective vital signs and subjective questionnaire data. This database structure also enables efficient storage, enhances data integrity, and supports a variety of analytical tasks for research purposes.

2 FIGURES

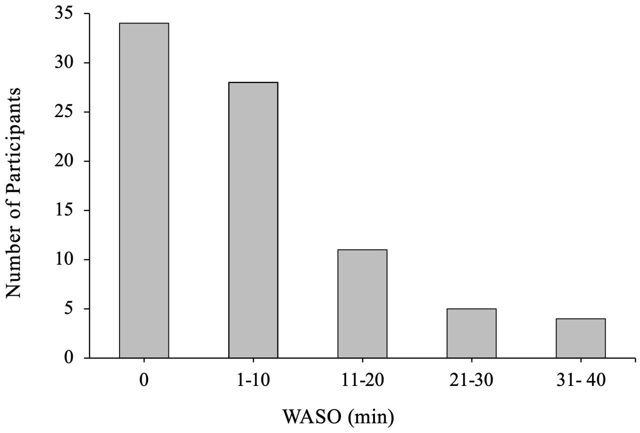

Figure S.2: Frequency distribution of WASO (Wake After Sleep Onset) categories among participants. The x-axis represents the defined WASO categories, and the y-axis represents the number of participants in each WASO category.

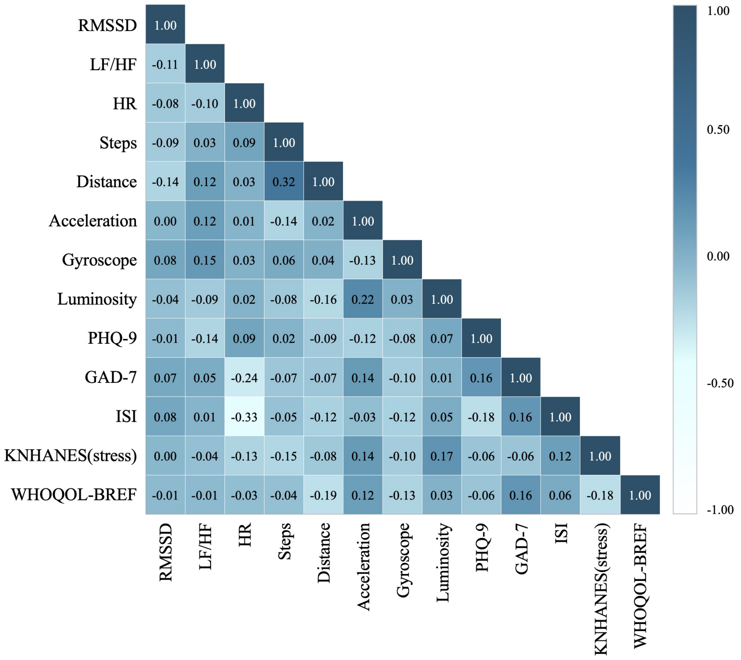

Figure S.3: Correlation Matrix of Biometric Signals. RMSSD, Root Mean Square of the Successive Differences; LF/HF, Low Frequency/High Frequency (an HRV metric); HR, Heart Rate; PHQ-9, Patient Health Questionnaire-9; GAD-7, Generalized Anxiety Disorder-7; ISI, Insomnia Severity Index; KNHANES, Stress Questionnaire for Korea National Health and Nutrition Examination Survey; WHOQOL-BREF, World Health Organization Quality-of-Life Brief Version.

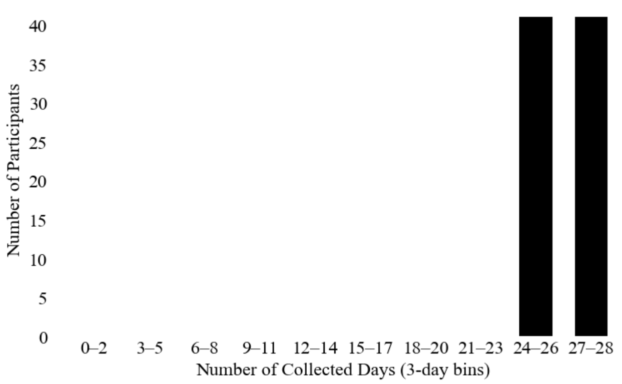

(A) Distribution of participants by valid days of sleep logs data collected.

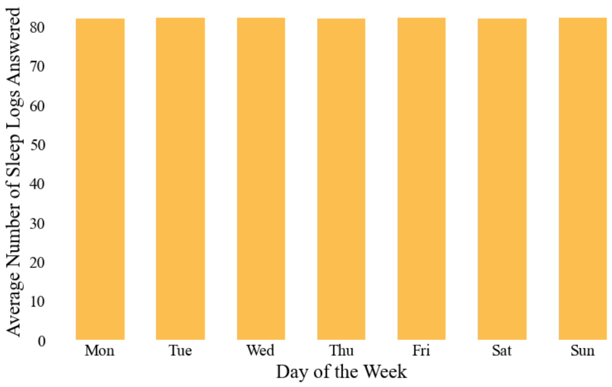

(B) Average number of sleep logs answered by day of the week.

Figure S.4: Overview of sleep log collection data. (A) illustrates the distribution of the number of valid days per participant contributing sleep logs data, binned in 3-day intervals. Note: the maximum number of valid days was 28 for the first experiment and 26 for the second. (B) presents the average number of sleep logs collected per day across the week. Note: each day of the week reflects the night of that day (e.g., Monday refers to sleep from Monday night to Tuesday morning).

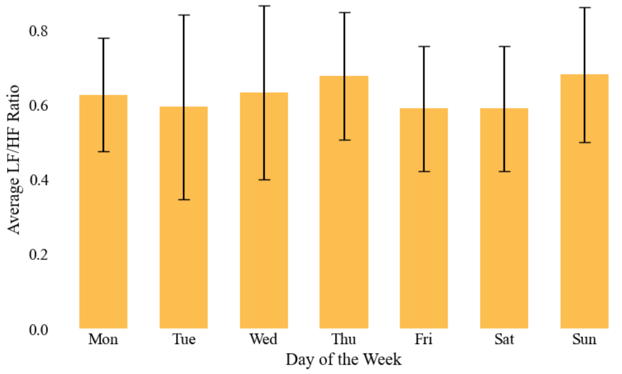

(A) Average number of LF/HF ratio measurements by day of the week.

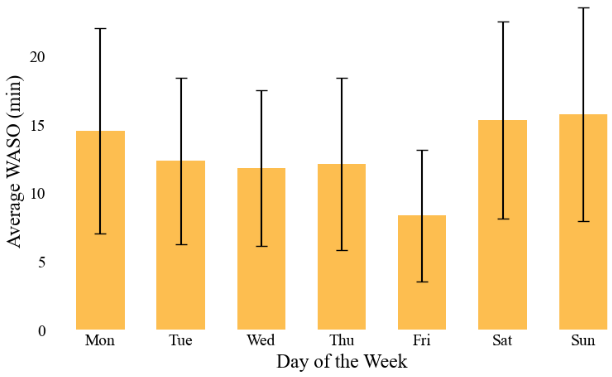

(B) Average number of WASO measurements by day of the week.

Figure S.5: Weekly summary of physiological and sleep indicators. (A) illustrates LF/HF ratio across days of the week, while (B) depicts the average WASO (in minutes) for each day. In both plots, the x-axis represents the day of the week, and error bars indicate the standard deviation across participants. Note: in the WASO plot, each day of the week corresponds to the sleep period from that night to the following morning (e.g., Monday represents sleep from Monday night to Tuesday morning).

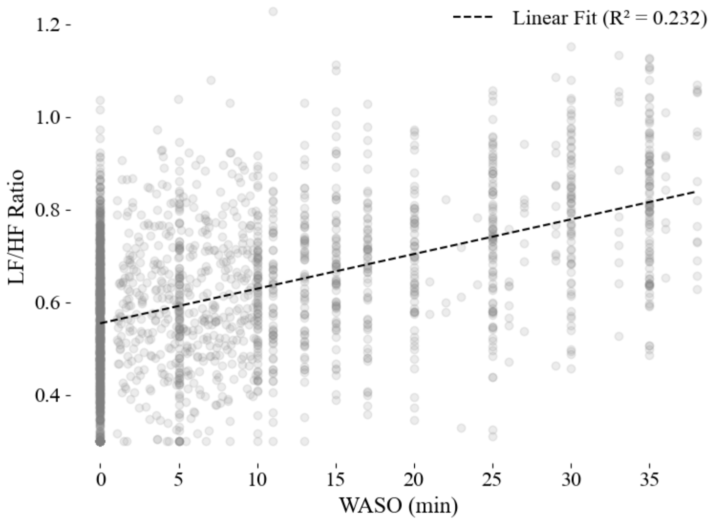

Figure S.6: Scatter plot illustrating the relationship between WASO values (in minutes) and LF/HF ratio. Each dot represents a daily sample from participants, with a dashed line indicating the linear regression fit to the data.

3 TABLES

Table S.1. Performance of the various models when predicting ISI (Insomnia Severity Index) and Sleep Time as the target variables (y), respectively, based on B_2, which is the best independent variable combination. Bold represents the best value, and underlining indicates the second best. The ISI and Sleep Time classification thresholds were determined based on their respective average values, and ARIMA classification thresholds were determined based on ROC curve analysis.

Feature	Model	Accuracy	Precision	Recall	AUROC	Loss
ISI	ARIMA	0.753	0.792	0.733	0.765	0.430
	Random Forest	0.781	0.801	0.765	0.784	0.422
	XGBoost	0.766	0.819	0.788	0.798	0.415
	GRU	0.722	0.800	0.692	0.745	0.399
	TCN	0.717	0.796	0.688	0.742	0.396
	Transformers	0.740	0.817	0.706	0.763	**0.380**
	LSTM	**0.832**	**0.822**	**0.793**	**0.815**	0.393
Sleep Time	ARIMA	0.680	0.671	0.688	0.692	0.420
	Random Forest	0.700	0.601	0.671	0.697	0.401
	XGBoost	0.711	0.640	0.682	0.704	0.395
	GRU	0.699	0.671	0.624	0.689	0.402
	TCN	0.681	0.662	0.610	0.676	0.417
	Transformers	0.678	0.669	0.603	0.672	0.422
	LSTM	**0.766**	**0.712**	**0.721**	**0.725**	**0.367**

Table S.2. Performance of the various models to predict WASO (as a binary classification) based on A_1, which is the second-best independent variable combination. Bold is the best value, and underlining is the second best. The classification threshold was determined based on ROC curve analysis.

Model	Accuracy	Precision	Recall	AUROC	Loss
ARIMA	0.752	0.801	0.781	0.779	0.416
Random Forest	0.775	0.786	0.764	0.771	0.417
XGBoost	0.781	0.805	0.748	0.783	0.419
GRU	0.721	0.790	0.690	0.726	0.410
TCN	0.718	0.778	0.686	0.713	0.415
Transformers	0.832	0.811	0.790	0.823	**0.408**
LSTM	**0.850**	**0.841**	**0.836**	**0.861**	0.410
